# Analysis of TACI mutations in CVID & RESPI patients who have inherited HLA B*44 or HLA*B8

**DOI:** 10.1186/1471-2350-10-100

**Published:** 2009-09-23

**Authors:** Manda L Waldrep, Yingxin Zhuang, Harry W Schroeder

**Affiliations:** 1 Allergy, Asthma and Immunology Clinic of Georgetown, Georgetown, Texas, USA; 2 Division of Clinical Immunology and Rheumatology, University of Alabama at Birmingham, Birmingham, Alabama, USA; 3 Division of Clinical Immunology and Rheumatology, University of Alabama at Birmingham, Birmingham, Alabama, USA

## Abstract

**Background:**

Recent reports have suggested that Common Variable Immunodeficieny (CVID) can present as an autosomal dominant trait dependent on the inheritance of a set of uncommon mutations/alleles of TACI (transmembrane activator and calcium-modulator and cyclophilin ligand interactor) involving exons 3 or 4. Penetrance, however, appears to be incomplete. Among our clinic population, the greatest genetic linkage for CVID is to the major histocompatibility complex (MHC) on chromosome 6. The majority of our patients have inherited HLA *DQ2, *DR7, *DR3(17), *B8, and/or *B44. Of these, HLA*B44 was present in almost half of the patients and was thus the most common susceptibility allele. HLA *B44 was also found to be over-represented among patients who presented to our clinic with adult-onset recurrent sinopulmonary infections (RESPI) and normal serum immunoglobulin levels, a cohort that included first and second degree relatives of patients with CVID. One of the two original reports of the association between TACI and CVID also reported Human Leukocyte Antigen (HLA) haplotypes. Of 13 affected subjects, nine had inherited HLA *B8 and six had inherited HLA B44. This raised the possibility that TACI mutations might synergize with MHC class I alleles to enhance susceptibility to humoral immune deficiency.

**Methods:**

We identified 63 CVID patients irrespective of HLA status and 13 RESPI patients who had inherited HLA*B44. To evaluate for mutations in the gene for TACI, we PCR amplified and sequenced TACI exons 3 and 4 from these patients.

**Results:**

Of the 76 patients, eleven proved heterozygous for a previously reported, silent T->G polymorphism [rs35062843] at proline 97 in exon 3. However, none of the 13 RESPI patients and only one of the 63 CVID patients inherited a TACI allele previously associated with CVID. This patient was heterozygous for the TACI A181E allele (exon 4). She did not carry *DQ2, *DR7, *DR3(17), *B8, or *B44.

**Conclusion:**

These findings suggest that TACI mutations are unlikely to play a critical role in creating susceptibility to CVID among patients with previously recognized MHC class I and class II susceptibility alleles.

Supported by NIH/USIDNET N01-AI30070, NIH R21 AI079741 and NIH M01-RR00032

## Background

In the United States, CVID is the most common primary immunodeficiency under the care of the clinical immunologist. CVID is a clinical diagnosis given to patients with unexplained pan-hypogammaglobulinemia in the presence of normal or near-normal B cell numbers. It has been called agammaglobulinemia with B cells to distinguish it from Bruton's agammaglobulinemia [1]. The most common clinical presentation is a history of recurrent pyogenic sinopulmonary infections [2,3]. Among Americans of European descent, it affects approximately 1 in 25,000 [4,5]. The frequency among Americans of African descent is twenty-fold lower [6]. These ethnic associations, coupled with the results of familial studies, have suggested that CVID has a strong genetic component [7,8]. However, identification of the causative genes has been difficult. It remains unclear whether the disease reflects a single Mendelian trait with incomplete penetrance, or whether it is the result of a combination of genetic lesions and environmental influences.

In our clinic in the Southeastern United States, the majority of patients with CVID have inherited part or all of two extended MHC haplotypes: HLA*DQ2,*DR7,*B44 or *DQ2,*DR3(17),*B8 [6]. These haplotypes are also common in patients with selective IgA deficiency (IgAD) [8-10]. In our population, hypogammaglobulinemia appears more closely linked to the class I locus than class II, with almost half of our patients inheriting HLA B44 [6].

In 2005, Johnston et al studied a series of patients who had presented to our clinic with a history of sinopulmonary infections (RESPI) similar in frequency and severity to that observed in CVID, but with normal serum levels of IgM and IgA and with IgG serum levels of 600 mg/dl or greater [11]. The distribution of MHC class I alleles in this RESPI population proved similar to CVID, with almost half of the patients inheriting HLA-B44. This led to the suggestion that RESPI might reflect a more moderate form of the same immune dysfunction that underlies the combination of recurrent infections and panhypogammaglobulinemia in CVID.

In the continuing search for genes that might contribute to the CVID phenotype, a number of investigators have focused on the study of candidates involved in either B cell proliferation or survival. In 2005, Salzer et al [12] and Castigli et al [13] reported an association between mutations in exon 3 or 4 in TACI and CVID. The complexity of the genetics of CVID and the role of TACI mutations is underscored by the recent report of discordance between the inheritance C104R mutation and CVID in a family with three affected siblings [14]. Interestingly, Salzer et al [12] had reported that of 13 affected individuals, nine had inherited HLA*B8 and six had inherited HLA*B44. This raised the possibility that joint inheritance of TACI mutations and HLA-associated susceptibility haplotypes might facilitate the development of immune deficiency and help explain variations in penetrance. To test this hypothesis, we examined TACI exons 3 and 4 among 63 patients with CVID and among 13 patients with RESPI from our clinic and tested for the joint presence of TACI and MHC-associated susceptibility alleles.

## Methods

### Materials

We identified 63 CVID patients irrespective of HLA status and 13 RESPI patients who had inherited HLA*B44. The clinical and genetic features of these populations have been previously described [6,11]. The study was performed under the guidelines of the Institutional Review Board of the University of Alabama at Birmingham. Informed consent was obtained from all of the study participants.

### HLA typing

Human Leukocyte Antigen (HLA) -B and -DR alleles were typed by PCR using LABType SSO PCR-SSO (Thermalcycler; Luminex Corporation, Austin, TX) at low resolution. Some patients were typed prior to the recognition of the HLA -DR17 split product, causing us to refer to HLA -DR17 as -DR3(17). We began to routinely perform HLA haplotyping in 2000.

### PCR amplification

We used the protocol of Castigli et al ([13]) to PCR amplify and sequence TACI exons 3 and 4 from DNA isolated from blood mononuclear cells.

## Results

### CVID

#### MHC haplotypes

Of the 63 patients with CVID, 23 out of 63 patients inherited HLA B*44 (36.5%) and 21 out of 63 (33.3%) inherited HLA*B8. Of these, 4 were compound HLA*B44 and HLA*B8 heterozygotes (6.3%). Seventeen patients had inherited at least one complete copy of the HLA*B8,*DR17(3),*DQ2 haplotype (26.9%), and seven had at least one complete copy of the HLA B*44,*DR7,*DQ2 haplotype (11.1%). One patient had inherited an entire copy of both of the haplotypes (UAB 00209). [Table 1]

**Table 1:** Characteristics of CVID patients with TACI exon 3 or 4 coding variants

		**HLA alleles**							
							
**TACI allele Patient Sample**	**Age at presentation**	**B**	**DR**	**DQ**	**Race/Gender**		**IgM g/dl**	**IgG g/dl**	**IgA g/dl**
**P97P**									
UAB00067	35	8,51	17,13	2,6	W	F	12	307	8
UAB00131	22	35,38	1,11	ND	W	M	5	312	1
UAB00256	49	38,51	4,11	3,3	W	F	4	69	1
UAB00021	54	44,61	3,2	2,5	W	M	34	339	17
UAB00245	35	44,44	7,15	2,6	W	F	53	314	8
UAB00604	54	44,58	4,8	4,7	W	F	27	525	55
UAB00111	32	ND	ND	ND	W	M	12	329	7
**A181E**									
UAB00142	35	39,62	8,13	4,6	W	F	33	279	8

#### TACI polymorphism

Seven of our patients were heterozygous for a silent T->G polymorphism (P97P, rs35062843) in exon 3. The allele frequency of 0.055 in this sample of patients was statistically indistinguishable from the frequency of 0.056 reported in dbSNP. Of these patients, 3 had inherited HLA B*44 and one patient inherited HLA B*8. In exon 4, we identified one patient (1.6%) who proved heterozygous for the A181E mutation. This patient, who was from a state adjoining Alabama, had inherited neither HLA *B44 or *B8. [Table 1]

The one patient who had inherited the TACI A181E allele presented to our clinic when she was 35 years old. She reported recurrent sinusitis, bronchitis, fatigue, and malaise. A chest x-ray revealed bilateral pulmonary nodules and mediastinal adenopathy. The physical examination revealed an enlarged spleen. The serum IgM was 28 mg/dl, the IgG was 256 gm/dl, and the IgA was reported as < 8 mg/dl. Her absolute T count was 1,109 cells per cmm with a CD4:CD8 ratio of 2.8. Her absolute B cell count was 140 per cmm. She had only 67,000 platelets per cmm of blood, and tests for anti-platelet antibodies were positive. She was determined to be HLA *DQ4, DQ6; -DR8, DR13, *B39, B62, and *A1, A3. She was placed on immunoglobulin replacement therapy supplemented with rotating prophylactic antibiotics. On 0.6 gm/kg of IVIG, her trough IgG reached 840 mg/dl, the size of her spleen markedly diminished, and the infections ceased. She reported a marked improvement in her sense of well being. Unfortunately, two years later she presented with a recurrence of splenomegaly, fatigue, and malaise. She underwent a splenectomy, but then proceeded to develop hepatomegaly, portal hypertension, pulmonary effusions, and ascites. A liver biopsy disclosed a polyclonal lymphocytic infiltrate. Lymphocytes were detected in the pleural and ascitic fluid. Cultures were negative, but she indicated symptomatic improvement after treatment with broad spectrum antibiotics. She returned to the care of her local physician who reported that her respite after antibiotic therapy did not last. She followed a progressive downhill course and ultimately expired. An autopsy was not performed.

### RESPI

#### MHC Haplotypes

We selected thirteen consecutive RESPI patients who had inherited HLA B*44 for study [Table 2]

**Table 2:** Characteristics of RESPI patients with P97P TACI coding variant

		**HLA alleles**							
							
**TACI allele Patient Sample**	**Age at presentation**	**B**	**DR**	**DQ**	**Race/Gender**		**IgM g/dl**	**IgG g/dl**	**IgA g/dl**
UAB00465	33	8,44	17,4	2,8	W	F	151	1460	425
UAB00463	34	8,44	17,7	2,2	W	F	133	757	221
UAB00183	37	44,27	11,15	6,7	W	F	76	718	225
UAB00020	48	44,35	7,15	2,6	W	F	153	754	105

#### TACI polymorphisms

In exon 3, we identified a silent T->G polymorphism (P97P) in 4 of our patients (30.8%). In exon 4, we identified no mutations. [Table 2]

## Discussion

Among patients with CVID, the frequent occurrence of selective to generalized antibody deficiencies among first or second degree relatives coupled with significant differences in prevalence among Americans of European versus African descent have provided strong support for the view that genetic inheritance plays an important role in the development of the disease[6]. However, only a minority of cases follow a simple Mendelian pattern of disease transmission. Among these families, loss of function mutations of key genes such as ICOS, CD19 and BAFF-R have offered a clear mechanistic rationale for antibody deficiency [2,3,5,15]. However, for the majority of patients with CVID, it remains unclear whether the disease is sporadic, the product of an environmental insult, the inheritance of a simple Mendelian trait with reduced penetrance, or a combination of one or more genes interacting with a chance environmental insult.

In 2005, replacement or frameshift mutations in the gene for TACI were identified in 13 of 162 CVID cases by Salzer et al [12] and in 4 of 19 cases by Castigli et al [13]. TACI (*TNFRSF13B*) is a member of the tumor necrosis factor receptor (TNFR) superfamily. It serves as a receptor for soluble B cell activating factor of tumor necrosis factor family (BAFF) and a proliferation inducing ligand (APRIL). These TNF-like ligands play key roles in the maturation and survival of B cells in the periphery. Within this population of patients, some of the probands carrying TACI polymorphisms demonstrated unique loss of function mutations. However, others were heterozygous for one of two single nucleotide polymorphisms (C104R and A181E), each of which created a replacement mutation in either exons 3 or 4, respectively. These and other studies identified additional mutations (e.g. S144X, R202H, ins204A), all of which were also found within exons 3 or 4 of TACI [16-18]. These mutations appeared to engender susceptibility to a form of selective or panhypogammaglobulinemia that was transmitted as a Mendelian autosomal dominant trait with incomplete penetrance [12,13].

Among the 13 patients reported by Salzer et al, nine had inherited HLA*B8 and six had inherited HLA*B44, which in our population are the two most prominent MHC alleles among our patients with CVID [12]. To us this raised the possibility of epistasis between a mutant TACI allele with altered function and an as yet unidentified gene or genes located near the MHC class I locus.

To test for this possibility of synergy, we evaluated 63 CVID patients irrespective of MHC allele and 13 RESPI patients who had inherited HLA *B44. Because multiple studies had identified mutations predisposing to CVID only within exons 3 and 4 [16-18], we focused our efforts on this region of DNA. We found a replacement mutation in TACI in only one patient. This provides support for the hypothesis that "the overall pattern of HLA types in individuals with TACI deficiency seems to be different than in individuals with idiopathic CVID" [12].

The CVID patient who was heterozygous for the A181E polymorphism did not inherit either of the MHC alleles that we have previously associated with IgAD or CVID in our population. The clinical course followed by this patient was more severe than what is common for our population, which supports the view that the A181E mutation places patients at a higher risk for complications and thus may warrant closer monitoring and evaluation [19].

The two original studies [12,13] reported a combined total of 17 cases with TACI replacement or frameshift mutations affecting exons 3 or 4 among 162 sporadic or familial index cases with CVID (9.3%). A follow-up study by Zhang et al [16] reported a frequency of 13 of 176 (7.3%) among sporadic cases alone. The frequency of 1 in 63 patients with a TACI mutation in exons 3 or 4 (1.6%) in our patient population is lower than for these previous studies (p = 0.056, χ^2^). It is possible that the inclusion of familial cases biased ascertainment frequencies. The majority of citizens of European descent in the state of Alabama and its surrounding regions are the descendants of a wave of immigrants from the states of Virginia, North and South Carolina, and Georgia that occurred between 1815 and 1840. Most of these pioneers were the descendants of peoples from Scotland, Ireland, Wales, and England that had migrated to the southern coastal regions of the United States in the previous century [20]. The low frequency of the A181E and C104R polymorphisms in our patient population may reflect genetic drift, founder effects, or both.

## Conclusion

In conclusion, our findings suggest that TACI mutations are unlikely to play a critical role in creating susceptibility to CVID among patients with previously recognized MHC class I and class II susceptibility alleles. The one patient we identified who inherited the TACI A181E allele had not inherited either HLA *B44 or *B8.

## Abbreviations

CVID: Common Variable Immunodeficiency; MHC: Major Histocompatibility Complex; RESPI: Recurrent Sinopulmonary Infection; HLA: Human Leukocyte Antigen; TACI: Transmembrane Activator and Calcium-modulator and Cyclophilin ligand Interactor; TNFR: tumor necrosis factor receptor; BAFF: B cell activating factor; APRIL: a proliferation inducing ligand; IgAD: IgA Deficiency

## Authors' contributions

YZ is a research associate in the lab of Dr Harry Schroeder who performed PCR amplification and sequencing of TACI exons 3 and 4 from DNA isolated from blood mononuclear cells. MW was an Allergy-Immunology fellow who analyzed the patient DNA sequences, interpreted data and drafted the manuscript. HWS conceived of the study, and participated in its design and coordination and helped draft the manuscript. All authors have read and approved of the final manuscript.

## Pre-publication history

The pre-publication history for this paper can be accessed here:


